# Primary care clinical practice guidelines in South Africa: qualitative study exploring perspectives of national stakeholders

**DOI:** 10.1186/s12913-017-2546-z

**Published:** 2017-08-29

**Authors:** Tamara Kredo, Amber Abrams, Taryn Young, Quinette Louw, Jimmy Volmink, Karen Daniels

**Affiliations:** 1 Cochrane South Africa, South African Medical Research Council, Cape Town, South Africa; 2 Centre for Evidence-Based Health Care, Faculty of Medicine and Health Sciences, Stellenbosch University, Cape Town, South Africa; 3 Physiotherapy Division, Faculty of Medicine and Health Sciences, Stellenbosch University, Cape Town, South Africa; 4 Deans Office, Faculty of Medicine and Health Sciences, Stellenbosch University, Cape Town, South Africa; 5 Health Systems Research Unit, South African Medical Research Council, Cape Town, South Africa; 6 Health Policy and Systems Division, School of Public Health and Family Medicine, University of Cape Town, Cape Town, South Africa

**Keywords:** Clinical practice guideline, Primary care, Qualitative interviews, Guideline development, Guideline implementation, Universal health coverage

## Abstract

**Background:**

Clinical practice guidelines (CPGs) are common tools in policy and clinical practice informing clinical decisions at the bedside, governance of health facilities, health insurer and government spending, and patient choices. South Africa’s health sector is transitioning to a national health insurance system, aiming to build on other primary health care initiatives to transform the previously segregated, inequitable services. Within these plans CPGs are an integral tool for delivering standardised and cost effective care. Currently, there is no accepted standard approach to developing, adapting or implementing CPGs efficiently or effectively in South Africa. We explored the current players; drivers; and the context and processes of primary care CPG development from the perspective of stakeholders operating at national level.

**Methods:**

We used a qualitative approach. Sampling was initially purposeful, followed by snowballing and further sampling to reach representivity of primary care service providers. Individual in-depth interviews were recorded and transcribed verbatim. We used thematic content analysis to analyse the data.

**Results:**

We conducted 37 in-depth interviews from June 2014–July 2015. We found CPG development and implementation were hampered by lack of human and funding resources for technical and methodological work; fragmentation between groups, and between national and provincial health sectors; and lack of agreed systems for CPG development and implementation. Some CPG contributors steadfastly work to improve processes aiming to enhance communication, use of evidence, and transparency to ensure credible guidance is produced. Many interviewed had shared values, and were driven to address inequity, however, resource gaps were perceived to create an enabling environment for commercial interests or personal agendas to drive the CPG development process.

**Conclusions:**

Our findings identified strengths and gaps in CPG development processes, and a need for national standards to guide CPG development and implementation. Based on our findings and suggestions from participants, a possible way forward would be for South Africa to have a centrally coordinated CPG unit to address these needs and aspects of fragmentation by devising processes that support collaboration, transparency and credibility across sectors and disciplines. Such an initiative will require adequate resourcing to build capacity and ensure support for the delivery of high quality CPGs for South African primary care.

**Electronic supplementary material:**

The online version of this article (doi:10.1186/s12913-017-2546-z) contains supplementary material, which is available to authorized users.

## Background

Since the first democratic elections in South Africa in 1994, there have been many initiatives aiming to transform the previously segregated and fragmented health sector [1, 2]. These include integrating the public health system at national level, shifting from racialised to integrated health departments; introducing a re-engineered primary health care (PHC) approach delivered through health districts; and, refocusing funding to priority programmes such as immunisation and HIV care [3]. Despite this, and the allocation of 8.5% of gross domestic product (GDP) to health care provision; South Africa’s health outcomes are relatively poor when compared to other middle-income countries with similar GDP percentage expenditure on health [1, 3, 4]. In the public sector, inequalities in healthcare delivery and outcomes persist between urban and rural settings and within and between provinces [5]. In response to this inequality and the high burden of disease from communicable and non-communicable causes, and violence and injury; the Minister of Health is driving forward plans for universal health coverage (UHC), as described in the National Health Insurance (NHI) White Paper [6–9].

Quality of health service delivery must be of high standard for UHC to achieve its intended goal [10–12]. Clinical practice guidelines (CPGs) are a common knowledge translation tool in policy and practice, covering clinical decisions at the bedside, governance of health facilities, health insurer and government spending, and patient choices. CPGs summarise rigorously gathered evidence, distilled for use by healthcare providers to standardise and provide best-available care [13, 14]. Within the range of operational tools available for policy implementation, CPGs are recognised as central to clinical service delivery [15, 16].

The South African government has introduced several PHC quality improvement programmes, including appointing District Specialist Clinical Teams (DSCTs) to support services within health districts. The ‘Ideal Clinic’ programme was initiated in 2013 to systematically consider and ensure adequate infrastructure, human resources, good governance and equipment for primary care [8, 17]. This programme is governed by specific clinical policies, protocols and CPGs, and aims to involve stakeholders across government departments, the private sector and non-governmental organisations (NGOs) to address health and social needs. In part, the intention is to integrate the preventative and curative health services in a patient-centred manner [18]. The ‘Ideal Clinic’, based on PHC principles, includes the Integrated Clinical Services Management programme, which focuses on efficient, cost-effective chronic disease management [17, 19]. In this context, CPGs are specifically used as clinical training tools for the DCSTs, and as part of clinical audit criteria to assess a facility’s attainment of ‘Ideal Clinic’ status [17].

South Africa has long contributed to CPG development with key players such as the National Department of Health (NDoH), clinical professional societies and NGOs producing guidance for their respective constituents. In particular, the NDoH’s Essential Drugs Programme has been producing guidance documents since the mid-1990s to support equitable use of medicines in the public sector. Other research on CPG development has been conducted in South Africa, in particular, exploration of policy development processes for maternal health and there has also been a quantitative evaluation of the quality of CPGs for PHC [20–23]. What is lacking, however, is comprehensive information on the processes, context and challenges for PHC CPG development and implementation in South Africa. The NHI system will require agreement and integration across sectors and jurisdictions, and national CPGs will need to speak to healthcare needs of all to ensure equal service delivery and redress of ongoing ‘fault lines’ in the system [5]. This transition from the current health system arrangement to NHI provides a window of opportunity to explore the current ‘state of play’ of CPG development and implementation in order to inform NHI processes.

Currently, there is no available guidance on standard approaches to developing, adapting or implementing CPGs efficiently or effectively in South Africa. To address this gap, the South African Guidelines Excellence (SAGE) project was established as a multi-partner research initiative aimed at setting in motion a stakeholder-driven process to contribute to the understanding of standards of national CPG development, adaptation and implementation [24]. The project consists of several components, including a mapping phase, development of an online CPG resource, and capacity building opportunities for those involved in CPG development, implementation and research. During the mapping phase, a cross-sectional analysis of a sample of 16 PHC CPGs was completed, identifying the strengths and gaps, in relation to global standards for CPG reporting [23, 25]. We found that overall AGREE II scores were poor to moderate, mostly due to poor reporting of rigour of methodological approaches; applicability; and, editorial independence. These findings are of concern as they may impact on the credibility of South African CPGs. In this paper we examine these issues in more depth by identifying the current role players; drivers; and the context and processes of PHC CPG development from the perspective of national stakeholders [26].

## Methods

This research makes use of qualitative methods, drawing on an interpretivist paradigm to explore national role players’ experiences in the processes of developing and implementing CPGs [26–28].

### Research context

South Africa has a population approaching 55 million, with a health system invested in primary healthcare and district level ownership [8]. Fiscal federalism is in place in which national government designs strategies, policies and clinical CPGs; and provincial governments implement CPGs, sometimes after adaptation, to the levels of healthcare from regional, district to community based health centres [2]. Although government is responsible for CPG development and implementation, many role players contribute to CPG activities to fill clinical guidance gaps, possibly duplicating, and possibly omitting key health areas.

### Sampling

Purposeful sampling began with consultations between the research team and CPG developers known to have a role in national CPGs [28]. Stakeholder groups included the NDoH, academics and researchers, specialist professional associations, medical schemes, NGOs, and the pharmaceutical industry. In addition, the five core NDoH-endorsed primary care CPGs were identified: Basic Antenatal Care, Integrated Management of Childhood Illness, the Essential Drug List (EDL) Standard Treatment Guidelines for primary care and Primary Care 101 (PC101), as well as a recently completed CPG for Health Promotion and these were used to guide sampling. We used a ‘snowballing’ approach in which a core group of individuals actively involved with CPG development or implementation in South Africa for primary care were invited to participate; these individuals were then asked to identify other ‘key role players’ for inclusion in the sample [28]. During the course of the study we recognised that some groups known to be involved with PHC service delivery or linked with the endorsed PHC CPGs had not been identified through snowballing. At interim analysis, the sample was dominated by doctors and pharmacists, thus, additional groups including allied health, dentistry, nursing, and nutrition were purposefully sought to fill gaps and achieve a satisfactory degree of representivity [28]. Those interviewed occupied senior managerial positions within government, academic or organisations.

### Data collection and management

Interviewers received training in semi-structured interviewing techniques from a senior social scientist researcher. All but one of the interviews were conducted in pairs. Interviewers were all female and included various professions including clinicians (medical doctors, allied health professionals), social scientists and basic scientists, all with previous interviewing experience, and in analysis of qualitative data. The lead researcher, present at most interviews, has experience, as a doctor and specialist clinical pharmacologist, in using and teaching CPGs. She is also known to some of those interviewed and is involved with CPG activities at academic institutions and government which may have facilitated access to some interviewees, but could also introduce response bias. For this reason, it was considered important to have two interviewers per interviewee, one of whom who was not engaged in CPG activity, and thus would have more distance so as to ensure objectivity.

Invitations were emailed to potential participants requesting a suitable time and venue for conducting interviews. Participants were provided with information sheets and consent forms prior to their interview. We aimed to interview everyone in person, but four participants requested telephonic interviews. Most participants chose to be interviewed at their place of work, with one exception who opted to do the interview at home. Two of those invited referred us to other colleagues to interview. None of those invited dropped out or requested to withdraw once we started. Interviews lasted 60 min on average (ranging from 40 to 90 min). One interview was spread over two sessions due to time constraints during the first meeting. Before commencing, the focus of the project and purpose of the interview were discussed, and consent was re-affirmed, thereby ensuring that participants understood clearly why they were selected, what the interview was about and what their rights were. The semi-structured interview guide (see Additional file 1) was tailored to the experiences of each interviewee and each interview built on the findings of previous interviews. In other words, as the interviews progressed, assumptions, queries, and gaps in the evolving data set were clarified through further data collection. Data saturation was achieved prior to final interviews. The additional 10 interviews were conducted to ensure a representative sample and that no new themes emerged.

Interviews were recorded using a digital recorder. All interviews were transcribed by a third party, blind to the aims of the study. Transcriptions were reviewed for accuracy by members of the research team, but not by those interviewed, making changes as needed based on the audio files. Data is stored electronically on password-protected computers; a masterlist and the consent forms are stored in a locked cabinet to which the study lead has access.

### Analysis

An iterative, thematic content analysis approach was used [29]. A team of four, initially deductively, read the first 24 transcripts to develop a coding list, maintaining a focus on the key research objectives, that is: players, process, context, drivers and barriers to CPG development or implementation [26]. Once this coding list was developed, a single researcher coded the remaining 13 transcripts independently. Coding lists were categorised by identifying common themes within the broad research question. All preliminary findings were presented and discussed with the team to verify further emergent themes at regular intervals until all interviews and analysis were completed (Additional file 2). A summary of the findings and emerging themes were presented to the larger project team – consisting of people who have experience in CPG development, PHC service delivery, and the South African healthcare context - for validation.

### Rigour

To ensure rigour [27], we initiated the project ensuring the question was *relevant.* Our study may be of interest to the national policymakers or other researchers, and given the paucity of information on CPG activities in middle or lower income settings generally, this may have global relevance. We ensured *validity* through detailed description of our approach to sampling, data collection, data management, analysis. We considered *reflexivity* as described in the methods and limitations of the manuscript. The results are most *transferable* to the South African context – where history and politics have impacted on policy development. However, some findings may be *transferable* to other developing country settings where transparency and CPG processes are also in transition. There may also be *transferability* within South Africa and learning from primary care CPGs to other CPG development.

### Ethics and reporting

The study was approved by the Research Ethics Committees of the South African Medical Research Council (EC002–2/2014) and Stellenbosch University (N14/02/008). All participants provided written informed consent. Names of participants were anonymised, however, it was explained to participants that despite efforts to maintain anonymity, their opinions might be recognisable in the reporting process. An opportunity for them to withdraw before or during the interview was provided (none took this opportunity), and to review how their opinions were conveyed in the final manuscript (some took this opportunity). We referred to the Consolidated criteria for reporting qualitative research (COREQ) reporting guidelines to ensure comprehensive reporting [30].

## Results

Overall, CPG development is a complex web of interactions between players and organisations, informed by values, politics and power. Values reported include distributive justice, standardising care and equitable access to medicines. The process for CPG production differs by setting and group and is often poorly articulated. Silos of guideline activity, both nationally and provincially, potentially result in duplicated efforts for the often volunteer teams of technical experts. There is recognition of a transition to more robust processes in some CPG development groups. There is also recognition and acceptance of the need to improve further to align with international standards; however, the financial and human resources are lacking.

We conducted 37 in-depth interviews with high-level policymakers and CPG contributors from national and provincial Departments of Health; professional societies; for-profit groups including pharmaceutical industry and medical schemes; university academics; funders and technical advisors (Table 1).

**Table 1:** Description of stakeholders sampled (37 total)

Background discipline	Medicine (19), pharmacy (5), nursing (4), allied health (3), dentistry (1), nutrition (2), non-clinical managers (3)
Provinces represented	Eastern Cape (1); Gauteng (16); Kwazulu-Natal (3); Western Cape (17)
Sectors and stakeholder groups	National (10) and Provincial Department of Health (2); Professional Societies (6); Private sector (pharmaceutical 1; and medical Schemes 2) (3); academia (14); non-governmental organisations (2)

### Role players in CPG development

#### Public sector players

Those interviewed agreed that NDoH plays the central role driving and developing guidance documents through their various directorates, supported by partners, including academic institutions, funders and technical advisors from multi-lateral organisations such as the WHO and UNICEF.

*Guidelines is a big national Department of Health role … [INT06].*

*It’s the government who is pushing, the government which defined the date, the approach and so on and as part of development partners, we go there to help. [INT23]*

Within government there is a central programme developing CPGs particular oriented to the essential medicines list which is linked with streamlining national medicine and device procurement; and in parallel there are disease or topic specific programmes, such as HIV, tuberculosis, rehabilitation and nutrition, developing CPGs, with potentially overlapping content.

In addition to the NDoH CPG development efforts, medical schemes, professional societies, at times with pharmaceutical industry support, NGOs, provincial hospital or clinic level initiatives all develop CPGs where no or limited guidance exists. Participants, generally academics from outside of government, suggested that their role in CPG development emerged to address gaps in clinical guidance, not covered by NDoH.

*But then some of the smaller, neglected diseases don’t have a directorate at the Department of Health or whatever ... And so often those are undertaken by professional societies [INT06]*

*Organisations like the TAC [Treatment Action Campaign] that has driven the engagement and enabled professionals to actually engage with government and get good policies in place and develop good guidelines [INT18]*

Furthermore, while PHC providers such as nurses play the central role in service delivery at PHC they are not seen to be driving CPG activities nationally, and were described as noticeably absent in leading roles except perhaps in consultation processes or external review of documents.

#### Private sector players

Private health insurance is an important player in clinical care in South Africa. Funding of medicines and devices is decided through advanced systems and committees within each scheme. Insurers are governed by the Council for Medical Schemes who develop the prescribed minimum benefit packages - however, updates to these have not been promulgated for more than a decade. Some participants described how the essential medicines programme should form the basis of the prescribed minimum benefit and ideally, aligning private sector with national government.

*Ultimately the view is to change the legislation with regard to private health care to make sure that this essential medicines list forms the basis of this minimum benefit package and then you remove a whole bunch of inequity out of this private health insurance* [INT11]

*At the end of the day, as we go into an NHI, this divide should not exist* [INT14]

The pharmaceutical industry, another private player, may contribute evidence to medical schemes, professional societies and government to encourage investment in their health technology. They provide grants to support some professional society guideline development. Beyond the guideline development they impact pricing of medicines, and influence practice of clinicians.

*We have over the years been very conscious of the role and the influence of industry in shaping decision-making, even within the various well-respected national societies [INT18]*

#### Consulting end-users and external groups

Although NDoH aims to involve various sectors and interest groups through workshops and consultation during CPG development, the need for tangible engagement from stakeholders was described as a key area for improvement.

*we had people from facilities, we had nurses at different strata, you had unions and you had nurses from facility level giving input to that scale-up plan which is very specific and thus you can say a guideline for getting your clinic to function optimally … we also involve the civil society organisations, our donor partners and the NGO’s that, we call them implementation partners funded by the donor partners, and then other government departments and also private sector organisations [INT27]*

*We tried, really tried by all means to involve all the stakeholders because with just us alone we won’t be able to, to reach you know everybody who really needs to be involved in addressing that particular problem … I do not think we have really found a better, you know, a better mechanism of really, you know, engaging the people who are, who are at the frontline of implementation on how do you really want us to package some of this document [INT37]*

### Values and drivers for CPG development

The dominant view expressed by participants was that CPGs are valued for supporting delivery of standardised, equitable healthcare, especially in the post-apartheid South African context to ensure access for all to PHC. CPG development was driven by the same complex influences as other health policies [31], despite different disciplinary and ideological backgrounds. According to our participants the values included commitment to the NDoH’s tagline “health for all” [INT04]; addressing historical inequalities; and standardisation of quality and cost-effective healthcare.

#### Driver: equity

The need for greater equity was raised in both public and private sector interviews. As a function of government, the NDoH’s Essential Drugs Programme, was viewed as promoting change from the earlier fragmented health systems arrangements and the principles of equity of access to medicines [32].

*the essential drugs programme basically brought together all the formularies from the different homelands throughout South Africa, and tried to level the playing fields to make sure that there is equity in the way medicines are made available and accessible … So we don’t want that disparity between provinces [INT16]*

Contrary to this, the medical schemes legislative environment was reported to encourage competition rather than collaboration which raised concerns regarding how this may perpetuate inequity and inappropriate spending.

*And we need to make sure our policy is fair, transparent and equitable within the context of the benefit designs that are … there are a lot of anomalies in private healthcare because it is hopelessly inequitable. Even the legislation with regard to private health care coverage is inequitable but we are sort of constrained within the sort of legislative environment that we operate in. And so we need to find a mechanism to be able to operate properly within that [INT11]*

#### Drivers: personal, fiscal or political interests

Although equity was a dominant driver, participants discussed the prevalence of personal, fiscal or political values or agendas amongst academics, the pharmaceutical industry or other commercial enterprises and international organisations. In addition, CPG development was described as being driven by priorities of funders including NGOs, international donors or industry for new research or products.

*One of the big issues … is who is driving its development and why. And if you think about why people develop guidelines, it’s actually the fact that a massive amount is driven by industry, and then it’s driven by the needs to some extent and personal interest to another extent. [INT20]*

*Everybody brings a bias to the table and WHO is a really sore point with a lot of our experts because they’re writing policy for …. Africa right, they’re writing public health policy ... If anybody should be, we should be telling WHO what to do [INT08]*

As CPG development is poorly funded, participants perceived this as an opportunity for the pharmaceutical industry to fund development through unrestricted educational grants, which in turn may undermine the independence and credibility of a CPG. However, this view was not shared by all within academia and professional societies, as in some cases limited funding and the needs of constituents were valued above potential conflicts of interest. It was also suggested bias was perceived as the norm rather than the exception in CPG groups within professional societies.

*we are critically dependent on drug companies. No one else is willing to fund guidelines. I mean, theoretically, if one looked at it in its purest form, the department of health should be funding all of this. They should be intricately involved – we should all be doing it together – but they don’t. They don’t at all [INT19]*

### Processes in place for CPG development

As we explored the deductive categories of players, drivers, context and process of CPG development, several sub-themes emerged including the perceived challenge of fragmentation of CPG development and implementation processes; human skills and resource shortages; and, gaps in standardised systematic methods.

### Fragmentation: socio-political environment impacts CPG processes

*So all are little silos inside other silos [INT20]*

Participants described fragmentation affecting both CPG development and implementation, with most expressing concern regarding inefficient use of limited human and funding resources. Fragmentation occurred between national departments, between the private and public sectors, between national and provincial departments of health, between provinces and within provinces and districts.

#### Fragmentation impacts development

The fragmentation within government programmes, and between the public and private sectors is described as predominantly affecting CPGs production. Silos within NDoH processes, where individuals work in closed teams, not communicating effectively across departments, teams or working groups, were thought to result in duplication, poor resource use and gaps in recommendations. For example participants explained that directorates may produce parallel guidance to that produced by another group or professional society. Some participants described improving coordination of CPG development processes, however, not consistently or universally. The slow progress in addressing known issues of fragmentation was attributed to the limited capacity (administrative, organisational) and inadequate financial resources from central government.

*There is nothing in the department of health that will take on community acquired pneumonia guidelines. Where does it fall? It doesn’t fall into any directorate. So the societies have traditionally done this [INT06]*

Poorly coordinated national CPG development is perceived to have knock-on effects as healthcare providers receive conflicting guidance, in turn hampering implementation.

#### Fragmentation impacts implementation

Participants described a disconnection between national and provincial government, suggesting this resulted in CPGs which lacked legislative power to enforce standardised implementation and impact service delivery. These responses reflects South Africa’s fiscal federal-oversight system, in which national government develops policies and provinces have independence to implement [33]. This form of governance may perpetuate disparity between provinces, however, there is recognised complexity given that each province has different infrastructure, governance strengths and capacity to adapt and implement policies.

*the whole national - provincial problem is a huge issue that this country has to kind of sort out … There is incredible frustration in that they [National Department of Health] don’t have the power and the provinces can kind of, they may say sort of set this policy but they can’t enforce it and no one is reporting, there’s no accountability [INT08]*

Despite concerns about differences between provinces, global best practice suggests that CPGs should be adapted to local contexts. South African provinces have vastly different contexts, including resources, cultures, and infrastructure that require context-specific adaptations. Thus, as expressed by some, there is an inherent tension in trying to develop CPGs as national standards, when needs might demand different regional or district approaches.

### Resource shortages: human capacity and time

Most participants expressed anxiety about lack of technical skills, dedicated time and funding for quality CPG development*.* This perceived ‘insurmountable task’ was thought to have a knock-on effects resulting in challenges with transparency and falling short of international standards.

*I sympathize with the department on a lot of levels because they don’t have the resources, they’re constantly fighting fires but now it’s just not appropriate anymore to have this, policy making should be transparent, it should be thought through, it should not be something done hurriedly [INT08]*

*As soon as you look at this process it becomes almost insurmountable. Essentially, what is international consensus on how it should be done, and then the reality is that there’s just a mass of work to be done to combat that load to be correct, even if you were just to perhaps redevelop from scratch, which is something you should probably do. And there probably isn’t the resources either in-house, or even if you were to spread it out, to get everything up and running at the same time [INT20]*

#### Human capacity shortages

Of the limited human resources, medical doctors appeared to dominate the national primary care CPG development groups, followed by pharmacists; with other disciplines working in parallel within different government directorates*.* Nurses were generally not part of national guideline groups.

*You know, there are very few people who can actually develop the guidelines from nursing. Very few people have that expertise – almost no one [INT18]*

Many individuals interviewed mentioned that volunteer clinical experts and members of guideline groups had multiple roles over and above their usual positions. They were often tasked with methodological work of searching for, appraising and synthesising evidence. The same experts may also be involved with CPG design and implementations. Some recognised this as a weakness, while others accepted this as the reality of low-resource environments.

*So they go through a lot of, it’s a lot of work, first of all, people who are doing other jobs [INT06]*

#### Lack of skills transfer in guideline groups

CPG development was seen by some as being dominated by the same individuals often included in CPG panels over many years. Limited capacity was felt to be, in part, a consequence of poor succession planning within government.

*I mean, you know, some of these folks have been involved forever [INT08]*

Some participants suggested that this hampered a hand-over of skills. More inclusive CPG panels were desired as an opportunity for ‘on the job’ capacity building, but at times, experienced members were lost prior to new members being adequately capacitated. Some participants were encouraged by the addition of new panel members – which they viewed positively as allowing the development of more transparent, inclusive processes.

#### Conflicts of interest: funding drives agenda

Participants suggested that when skills to conduct the necessary technical work are deficient, groups with financial or other vested interests, often linked with pharmaceutical industry, may use the opportunity to drive their interests for marketing their health technologies. A lack of capacity to synthesise evidence could result in a reliance on the industry for support. Pharmaceutical players have resources to package evidence or fund CPG activities. The implication is due to the resource shortages described the industry may exercise their influence.

*Now that process is terrible because a lot of the clinical colleagues do not have the skills to put an evidence based discussion together, so that’s where a lot of the conflict comes in. They get the industry to write them [INT01]*

### Gaps: lack of systems for CPG development

#### No standardised processes for development

CPG development is not uniformly organised within the different NDoH programmes.

*It’s chaotic, it’s uncoordinated, it’s opaque [INT08]*

Participants suggested some programmes had structured systems while other programmes’ processes were perceived as “ad-hoc” or “chaotic” or in the case of private sector, guided by outdated protocols.

*Well, I don’t know what happens. They seem to be like a complete black box [INT03]*

*told by the minister, you know, like we need these guidelines out immediately. And then you’ll get stuck into it, and then there will be a new HoD [head of department], and then there will be a new this and there will be a new that. You know, the process is just very, very chaotic locally [INT14]*

*For instance, like the PMB [prescribed minimum benefit] guidelines, the algorithms are really a mess at the moment. They were published 15 years ago or whatever, and they are just a little one-pager with a few little lines. And they were extensively updated five years ago…. and they are still not promulgated. The private sector is sitting in a vacuum because the medical aids by law only have to fund to that level [INT18]*

Some CPG development groups such as the NDoH Essential Drugs Programme, established with good intentions during the post-apartheid period, were described as improving their processes over time; having put in place rigorous CPG development approaches. They were also criticised for poor communication and lack of transparency in decision-making. This group has explicit documents for ensuring interests are declared and confidentiality respected [34]. What is not apparent in the available documents is how transparency can be improved and processes shared to build public trust. Participants from outside government tended to have less trust in the government processes due to unsatisfactory experiences with trying to contribute to the CPGs which seemed to lead to reluctance to buy-in from some individuals and groups.

*I mean the EDL seems like a very well-run process. Now I mean I have issues with the EDL not being transparent, I mean I think I would like to see minutes, I think as public sectors, you know, public funds and that sort of thing, we’re entitled … [INT08]*

*And I think we have to really consider what the best means is of documenting evidence and then sharing that evidence in order to get buy-in [INT21]*

#### Managing conflicts of interest

Our academic participants often held multiple roles, both for government and their institutions. Those involved with professional societies reported their desire to collaborate with or be endorsed by government. Some societies seemed to be successful in working with government or identifying independent funding to develop CPGs, however, most were described to rely on pharmaceutical companies. Some members of professional societies were described as receiving funding from many industry sources, but the processes for reporting or managing these potential conflicts differed.

*Everyone is going to have a conflict of interest, because everyone is going to have to have received funding from someone for something ... So the answer is probably yes, we should, but I think at the end of the day it’s probably not practical [INT19]*

### Transition to better processes

Participants indicated that improvements are needed for CPG development to meet global standards, however, some reported that over the past 20 years there has been slow but persistent progress and a shift to increasingly transparent systems and methods for CPG development.

*I think guidelines have come a long way, I think they’re much more evidence based [INT06]*

*There’s a keen awareness that it could be done better and that there should be some sort of debate [INT21]*

The commitment of CPG developers to advance CPG processes is an influential enabler of continued progress.

### Implementation processes lag behind

Several participants complained that CPG implementation is lagging and requires additional specific skills and adequate funding to ensure recommendations reach end-users and contribute to improved patient outcomes.

*in terms of guideline development there’s still a lot of work that needs to be done, but in terms of implementation there’s more work that needs to be done [INT16]*

For the most part, national CPG developers, had fewer responses to questions regarding implementation, and referred us to provincial players to explore this further.

## Discussion

Global reporting for CPGs requires adherence to several quality standards including a description of a clear scope for the CPG; inclusion of all relevant stakeholders in development; rigorous methods for finding and assimilating evidence; and ensuring conflicts of interest and funders interests are recognised and managed [14, 35]. Yet there is evidence that South African CPGs fall short of quality reporting standards [23, 36]. We sought to explore the reasons behind this through the perspectives of national stakeholders regarding current processes, drivers, enablers and barriers for primary care CPG development. Our analysis suggests that the context and processes for CPG development represent a complex network of interactions, informed by values and power. There are multiple stakeholders, across government departments, healthcare disciplines, and sectors contributing to CPGs with varying skills and intentions. The NDoH is the key role player in CPG development for the public sector, with professional societies and other organisations filling in gaps (i.e., topics where guidelines do not exist, or in situations where guideline updates have not been undertaken for a number of years). Despite the common view that CPGs are valued for supporting delivery of standardised, equitable healthcare, there is also a belief that CPGs may in some instances be manipulated by commercial, personal or other interests.

CPGs aim to address health inequity as reflected in the national policies and plans for PHC reform. CPGs are specifically mentioned as useful tools to assist several key programmes including the NHI and the ‘Ideal Clinic’ [15]. In light of the intended transition to UHC through the proposed NHI funding system, private and public-sector CPGs will need to be aligned to ensure equitable access to quality healthcare services [7, 9]. In this context, concerns were raised regarding the parallel private and public healthcare system wherein private insurers operate independently from national government. The out-dated clinical protocols and Prescribed Minimum Benefits packages mean that private sector funders have freedom to drive healthcare decisions based on criteria other than best evidence or cost-effectiveness. The current proposal for NHI recognises this deficiency, and proposes revision of the medical schemes Prescribed Minimum Benefits and for health technology assessment to underpin clinical recommendations [9].

### Processes for CPG development in SA

The slow progress experienced in improvements in the health system and the fact that issues like fragmentation still persist has been highlighted in other health systems research [5]. Despite the introduction of key health policy reforms [5, 37], historical issues like fragmentation, human resource challenges and paucity of standardised systems continue to hamper progress in all areas of healthcare delivery [38]. Interviewees consistently reported concerns about the fragmented health system and its impact on CPG development and implementation. Fragmentation of CPG development may be understood to reflect these longstanding weaknesses in available systems resulting in opaque methods for CPG development; possible duplication due to lack of central oversight and communication between national CPG development groups; under-resourcing of people with specific skills to develop and implement CPGs; and, gaps in feedback and communication systems between stakeholders.

Several well-credentialed international groups have developed standardised methods outlining key steps to ensure trustworthiness of the final CPG [13, 39, 40]. We found that no such guidance exists in South Africa, and most interviewees described ad hoc processes, including inadequate consultation, and poorly managed conflicts of interest which may result in biased CPG recommendations, and diminished buy-in for CPGs [41]. These deficits may, in turn, impact on implementation. Implementation particularly was an issue of the national-provincial disconnect, however, only few of those interviewed were closely involved with implementation as this is largely the responsibility of provincial government.

Lack of adequate Human Resources for Health is a commonly reported problem in South Africa, particularly in rural districts [2, 5, 42]. For CPGs, we found that there is a limited pool of skilled contributors to CPG development, who are often working voluntarily or for limited remuneration and are overburdened. The specific technical skills gap identified includes capacity to synthesise and incorporate evidence; regular communication on CPG processes with stakeholders; and design and implementation for CPGs.

### Limitations and strengths

Limitations of our study include potential for sampling bias. Our sample is dominated by medical professionals, many from Gauteng and Western Cape Provinces. Our sample probably reflects the reality of skewed power dynamics in CPG development in South Africa, where many who lead national knowledge production, and therefore are able to contribute their voluntary time to the process, may be based in the Western Cape and Gauteng. To minimise and mitigate this potential bias, we allowed for snowballing for any and all national contributors to CPG processes; in addition, we purposively sampled across all primary care disciplines. The skew sample suggests that CPG development needs to become more inclusive of different disciplines in different provinces throughout South Africa. Another possible limitation is response bias as those interviewed are all active members in CPG development and likely to be positively inclined towards the value of the work. Therefore, we explored the players, drivers, processes and context rather than the perceived value of CPGs. Another potential limitation is the impact of where researcher team members are ‘situated’, their institutional affiliation, and perceived credibility also called ‘positionality’ [31]. Positionality may facilitate gaining access to policymakers and discussion of sensitive issues and it may allow for interpretation of nuanced cues, but may also skew responses, similar to the Hawthorn effect [28, 31]. Generally one of the interviewers was from Cochrane South Africa, a recognised specialist unit for evidence synthesis. However, the senior experienced policymakers and CPG contributors interviewed were thought to be peer level to the interviewers and less likely to be influenced by power dynamics commonly ascribed to interviewer/interviewee relationships [28].

Our study also had several strengths such as the teams’ prior training in qualitative interviewing; pre-knowledge of the CPG context, augmented by speaking to experts; the inclusion of members from different disciplines who could point to gaps in the process, thus enhancing rigour; and, reaching a range of participants, including senior members of the NDoH.

### What does it mean for SA CPG developers and development?

We found overwhelming commitment by those involved and slow but consistent transition to improving systems and processes. Commonly shared values regarding addressing inequitable clinical service through improved access to medicines and care defined by good quality CPGs may serve as an enabler to further processual improvements. Based on our findings, we feel that the key areas requiring attention include the need to reduce fragmentation by considering central coordination of CPG activities with buy-in from public-private stakeholders and ongoing communication between stakeholders already involved. We recommend that this be underpinned by agreed national standards and processes for CPG development and implementation, considering different provincial contexts. Finally, we feel that resourcing of activities is key to develop capacity to conduct methodological work, support clinical recommendation decision-making using transparent processes and improve communication, dissemination and implementation.

## Conclusion

As South Africa transitions towards the NHI system and the ideal of “health for all” there is an opportunity to reflect on lessons learned from PHC CPG contributors, and build on global experience and knowledge. WHO’s PHC Alma Alta Declaration states that PHC is a fundamental right and that ‘primary health care is essential health care based on practical, scientifically sound, socially acceptable methods and technology’ – in our context, CPGs provide the bridge and process through which this may happen [19]. However, South Africa is among many countries with faltering progress in advancing principles of WHO’s Alma-Ata for universal access to PHC [19, 38]. The data suggests that the current parallel private-public health systems pose a substantial challenge to uniform healthcare access. Participants describe commitment on the part of government, and those who support government in their CPG endeavours, to build collaborative, transparent, adequately funded and staffed systems that foster communication and encourage efficient use of the country’s scarce resources. A national CPG coordination unit could assist to develop credible, efficient structures to address the challenges identified.

## Additional files

Additional file 1: Interview schedule for semi-structured interviews (Table of questions asked to participants). (DOCX 13 kb)

Additional file 2: Analysis – Sub-themes for theme context (Codes generated from the analysis). (DOCX 16 kb)

## Abbreviations

COREQ: Consolidated criteria for reporting qualitative research
CPG: Clinical practice guideline
DSCT: District specialist clinical teams
EDL: Essential drug list
GDP: Gross domestic product
HOD: Head of department
NDoH: National department of health
NGOs: Non-governmental organisations
NHI: National health insurance
PC101: Primary care 101
PHC: primary health care
PMB: Prescribed minimum benefit
SAGE: South African Guidelines Excellence project
TAC: Treatment action campaign
UHC: Universal health coverage
