# Supplementary material for: Primary care clinical practice guidelines in South Africa: qualitative study exploring perspectives of national stakeholders
Source: BMC Health Serv Res. 2017 Aug 29;17:608. doi: 10.1186/s12913-017-2546-z (PMC5575947; doi:10.1186/s12913-017-2546-z)
Supplement: Supplementary file 1 — Interview schedule for semi-structured interviews (Table of questions asked to participants). (DOCX 13 kb) [file 12913_2017_2546_MOESM1_ESM.docx]

# Additional file 1. Interview schedule for semi-structured interviews

| BACKGROUND QUESTION: what is your experience and understanding of what a guideline is or does? |
| --- |
| 1. What is your context (role, position) as it links to clinical practice guidelines? |
| 1. What processes of primary care clinical practice guideline development, contextualisation, adapting, and implementation are in place? |
| 1. Who is involved/ role players? |
| 1. What works for clinical guideline development? What could be better? (if relevant to the informant) |
| 1. What works for clinical guideline implementation? What could be better? (if relevant to the informant) |
| 1. If we want to know more, who should we speak to? |
