# Supplementary material for: Primary care clinical practice guidelines in South Africa: qualitative study exploring perspectives of national stakeholders
Source: BMC Health Serv Res. 2017 Aug 29;17:608. doi: 10.1186/s12913-017-2546-z (PMC5575947; doi:10.1186/s12913-017-2546-z)
Supplement: Supplementary file 2 — Analysis – Sub-themes for theme context (Codes generated from the analysis). (DOCX 16 kb) [file 12913_2017_2546_MOESM2_ESM.docx]

Additional file 2. Analysis – theme: context

| Adapt |
| --- |
| Agenda setting |
| Assessment in some clinics - building evidence to inform circulars |
| Audience |
| Benefits of using guidelines |
| Brazil - community health team model |
| Budget managing - pooled resource used best |
| Budgeting for input into guidelines |
| Buy in - process |
| Buy-in - stakeholder |
| Champions |
| Change - curriculum |
| Collaboration |
| Committee membership selection |
| Communication/Transparency |
| Complexity |
| Complexity of decision making |
| Components of guideline |
| Composition of guideline committee |
| Conflict of interest |
| Consensus reaching |
| Context |
| Critical engagement lacking |
| Defensive against guidelines |
| Deference to EDL over other subcomittee |
| Definition of guidelines |
| Description of med aid guideline committee members |
| Differences |
| Disagreement |
| Dislike of guidelines |
| Dissemination and uptake |
| DoH Ignores the process |
| DoH Relationship w/WHO |
| DoH slow to update and respond |
| EDL |
| Engagement |
| Evidence |
| Fluidity of evidence |
| Focus |
| Fragmentation and Silos |
| Frustration with DoH |
| Funding |
| Gatekeeping |
| Good Communication with DOH |
| GP DoH under Administration |
| Guideline definition - consensus needed |
| Guideline importance |
| Guideline process vetting |
| Guideline purpose |
| Guidelines - critique |
| Guidelines and med aids |
| Guidelines as evidence for motivation for benefits |
| Guidelines as guides, not mandated procedure |
| Guidelines as job aids |
| Guidelines can guide research |
| Guidelines cannot replace the art of medicine |
| Guidelines -context needed |
| Guidelines define interactions |
| Health Scheme relations with Guidelines |
| Historical context |
| Human capacity |
| Industry |
| Landscape |
| Legal |
| limits of guidelines use |
| Med Aids |
| Membership - committee |
| motivation |
| NPO input |
| Nuances in med aids |
| Old Boys |
| Outdated guidelines inform minimum standards |
| Overlap guideline efforts |
| Personal context |
| Philosophy develoment not cookbook |
| Players |
| policy sets standards |
| Politics |
| Pragmatic |
| Primary health care and patient responsibility |
| Process |
| Prof Society |
| Province differences |
| Province V NDoH |
| Public V Private |
| Quality of care defined |
| Resources limited |
| Responsible resource distribution |
| Rx Availability - only that on the guideline |
| Silos not collaborating - information and efforts being repeated |
| Society produced guidelines |
| Staff in specialized silos |
| Staffing committees |
| Stakeholder engagement |
| Stakeholder input generation as important part of the process |
| Technology assessment |
| Tension in population level approaches and indiv patient needs |
| Thankless |
| Transparency |
